# Metabolic Complications of Chronic HIV Infection: A Narrative Review

**DOI:** 10.3390/pathogens11020197

**Published:** 2022-02-01

**Authors:** Nathan W. Cummins

**Affiliations:** Section of Infectious Diseases, Mayo Clinic College of Medicine and Science, Rochester, MN 55905, USA; cummins.nathan@mayo.edu; Tel.: +1-(507)-284-3747

**Keywords:** HIV, cardiovascular disease, dyslipidemia, diabetes mellitus, osteoporosis

## Abstract

As persons who are HIV positive and on suppressive antiretroviral therapy live longer, there is increased incidence and recognition of several metabolic complications of this chronic infection. These metabolic complications of HIV infection can result from the infection itself and/or otherwise effective antiviral treatment and can have significant impacts on morbidity and mortality. Some metabolic complications of HIV infection are preventable but most are modifiable, and therefore, active surveillance and screening are warranted. The purpose of this narrative review is to highlight the most common metabolic complications of chronic HIV infection, associated risk factors, diagnosis, and management.

## 1. Introduction

Forty years into the HIV pandemic, the once universally fatal infection has been transformed into a chronic, manageable medical condition due to highly effective antiretroviral therapy, which can suppress active viral replication for decades. Persons living with HIV (PLHIVs) on suppressive antiretroviral therapy can have a nearly normal life expectancy, even after adjusting for confounding comorbidities, such as hepatitis co-infection, drug or alcohol use, or smoking [1]. However, even treated chronic HIV infection comes with an expected life span deficit of 6 to 14 years [1] due to the presence of HIV-associated, non-AIDS conditions. These conditions are referred to as metabolic complications of HIV infection for the remainder of this review.

The pathophysiology of the metabolic complications of chronic HIV infection is multifactorial. Complex interactions within the host between ongoing low-level viral replication, chronic inflammation, accelerated aging, and long-term adverse effects of antiretroviral therapy drive the development of these complications. These complex interactions have been summarized in the term “inflammaging”, which associates immune system dysregulation with the development of metabolic complications through a process of accelerated biological senescence [2]. On a phenotypic scale, this inflammaging in chronic HIV infection is associated with increased frequency and severity of various age-related morbidities, including immunosenescence, cardiovascular complications, age-related malignancies, cognitive decline, frailty, low bone mineral density (BMD), and fragility fractures [3]. This inflammaging, however, is apparent also at the molecular level. Epigenetic studies of DNA methylation in peripheral blood mononuclear cells (PBMCs) from PLHIVs reveal advanced molecular age compared with chronological age to a similar degree (5–10 years) as the persistent life expectancy deficit seen in these patients [4].

Below, we will several metabolic complications of chronic HIV infection, including cardiovascular disease (CVD), dyslipidemia, fat redistribution syndromes, insulin resistance, and bone disease. 

## 2. Cardiovascular Disease

According to the World Health Organization, ischemic heart disease and stroke continue to be the two leading causes of death worldwide and are increasing in incidence [5]. This is most likely due to a globally increasingly aging population with reduced mortality from communicable diseases and worsening lifestyles. These same factors and phenotypic results are apparent and exaggerated in PLHIVs. The epidemiology of cardiovascular disease in PLHIVs has been studied extensively and reviewed elsewhere [6], with a clear and consistent relative risk increase of between 1.2 and 2.1 for acute myocardial infarction, diastolic heart dysfunction, and stroke for PLHIVs compared with persons who are HIV negative, and age- and sex-matched controls. This is consistent in women and persons with viral hepatitis co-infection. However, it should be noted that the absolute risk increase in most studies is low, as most studies are conducted in younger populations and are confounded by a high prevalence of other modifiable CVD risk factors (smoking, etc.).

CVD risk is increased in PLHIVs through a combination of factors, including effects the virus and its treatment. Chronic HIV infection is characterized by both abnormal immune activation and hypercoagulability [7], manifested by increased expression of CVD biomarkers such as interleukin 6, C-reactive protein, D-dimer, and Von-Willebrand’s factor (vWF). There is evidence of endothelial dysfunction, with impaired arterial relaxation and increased expressions of monocyte chemoattractant protein 1 (MCP1), P-selectin, vascular cell adhesion molecule 1 (VCAM), vWF, and tissue plasminogen activator (t-PA). Finally, there is the possibility of direct viral induced endothelial and cardiomyocyte injury from viral proteins such as Gp120 [8] and Tat [9]. 

Conversely, antiretroviral therapy may contribute to increased CVD risk through medication induced dyslipidemia or insulin resistance (discussed below). This was reflected in an increased rate of CVD in PLHIV on antiretroviral therapy compared with persons who are ART naïve in the D:A:D (Data Collection on Adverse Events of Anti-HIV Drugs) Study Cohort [10]. On the other hand, structured treatment interruptions of ART in the SMART (Strategies for Management of Antiretroviral Therapy) trial were associated with increased risk of cardiovascular events compared with continuous viral suppression on therapy [11]. These different study outcomes were possibly due to acute metabolic derangements associated with either new adverse drug effects or rebound viremia. 

## 3. Dyslipidemia and Management of CVD Risk

Dyslipidemia is common in PLHIVs, occurring in up to 40% of individuals [12]. In the pre-HAART era, this mostly took the form of reduced high-density lipoprotein (HDL) and low-density lipoprotein (LDL) levels with elevated triglycerides. However, class- and drug-specific ART adverse effects have changed this profile to include various degrees of elevations in LDL and TG levels, with equivocal changes in HDL. HIV protease inhibitors (PIs) typically raise both total cholesterol and triglycerides, although to a lesser extent with darunavir and atazanavir, whereas non-nucleoside reverse transcriptase inhibitors (NNRTIs) and integrase inhibitors (INSTIs) typically result in total cholesterol elevations more than hypertriglyceridemia. 

Management of excess cardiovascular risk in PLHIV focuses on risk stratification and modification of risk. The use of standard CVD risk estimators is recommended without modification for HIV status [13]. Routine measurement of fasting lipids and management of dyslipidemia according to established guidelines in the non-HIV population is recommended, along with counselling on exercise, diet, and smoking cessation. Careful management of comorbid diabetes mellitus and hypertension are essential. 

The management of hypercholesterolemia is accomplished predominately with statins. Statins have significant CYP3A4 interactions with PIs, which increase the concentration of statins, increasing the risk of adverse events such as rhabdomyolysis. The co-administration of PIs with simvastatin or lovastatin is contraindicated. Atorvastatin, low-dose rosuvastatin, or pravastatin for mild LDL elevations are preferred in this setting. NNRTIs decrease the concentration of statins, resulting in decreased efficacy. Hypertriglyceridemia is managed with fibrates, which are metabolized through CYP4a and have no significant drug interactions with antiretrovirals. Combination therapy regimens with niacin, fish oil, and ezetimibe are sometimes necessary but safe. 

The role of CVD risk in ART selection is controversial in the post-PI era. NRTI abacavir use was associated with increased risk of AMI in the D:A:D cohort in 2008. Conflicting results have occurred since then in several observational studies and pooled results from RCTs. Abacavir was initially removed as a first line agent in 2008 by the US Department of Health and Human Services (DHHS) for these reasons but was reinstated back as a recommended agent in 2015 in co-formulated tablets (DTG/ABC/3TC) [14]. 

## 4. Fat Redistribution Syndromes

Changes in lean body mass and fat distribution can occur in nearly half of PLHIVs [15] and are both cosmetically disconcerting for PLHIV and markers of increased CVD risk. Wasting, or loss of lean body mass, was more prevalent in the pre-HAART era but still occurs in chronically untreated persons. Lipodystrophy syndromes were a development of the post-HAART era and manifest as peripheral lipoatrophy, central lipohypertrophy, or a mixed picture. Risk factors for lipoatrophy include advanced age; lower baseline CD4 T cell count; higher viral load; coinfection with hepatitis C; or treatment with NRTI thymidine analogues, such as stavudine or zidovudine. These agents result in NRTI-induced inhibition of mitochondrial DNA polymerase-γ. Risk factors for lipohypertrophy include advanced age, female sex, increased baseline body fat, duration of ART, and poor diet. Lipohypertrophy can been seen with PI-based and NNRTI-based ART regimens. While the underlying mechanism is unclear, some in vitro studies suggest that these medications increase the adipogenic differentiation of mesenchymal stem cells [16]. 

Clinical management of HIV-associated lipodystrophy syndromes is often disappointing. For both syndromes, the ART regimen switch from offending agents if possible is recommended. Thiazolidinediones have mixed clinical trial results in the management of lipoatrophy and carry significant risks of CV safety and hepatotoxicity. Surgical management of lipoatrophy with gel fillers or autologous fat transplantation is not often cosmetically satisfactory. Lipohypertrophy is primarily managed through diet, exercise, and metformin if there is evidence of insulin resistance. Tesamorelin—a recombinant human growth-hormone-releasing hormone—decreases visceral adipose tissue, improves lipid profile, and has no effect on insulin resistance; however, rapid re-accumulation on discontinuation has been noted in clinical trials, and there are no long-term safety data. Suction-assisted lipectomy may be used for lipohypertrophy. 

## 5. Insulin Resistance and Diabetes Mellitus

Often concurrent with other manifestations of metabolic syndrome, including dyslipidemia, lipodystrophy syndromes, and cardiovascular risk, are insulin resistance and type 2 diabetes mellitus in PLHIVs [12]. Clinical risk factors for insulin resistance in PLHIVs include advanced age, obesity, non-white race, family history, HCV coinfection, stavudine or PI use, and presence of fat redistribution. PIs inhibit the GLUT4 glucose transporter [17], but this represents only one specific mechanism contributing to insulin resistance in PLHIVs. A more complex interplay of genetic predisposition, chronic inflammation, accelerated age-related insulin resistance, and hepatic dysfunction associated with HIV are likely involved. As with dyslipidemia, frequent screening of fasting glucose and pharmacologic management per guidelines in the non-HIV population are recommended. 

## 6. Bone Disease

PLHIVs have an increased incidence of multiple phenotypes of bone disease, including osteopenia, osteoporosis, and osteonecrosis, resulting in increased rates of clinically significant traumatic and non-traumatic fractures, occurring in up to 7% of individuals [18]. There are multiple contributing factors involved, including increased prevalence of traditional risk factors for bone disease in PLHIVs, chronic inflammation, low body weight, hypogonadism, vitamin D deficiency, IVDU, and HCV coinfection. Nearly all antiretrovirals have also been linked to bone loss, although tenofovir disoproxil fumarate (TDF) and PI use have been most studied and associated with bone loss. Screening with dual-energy X-ray absorptiometry (DEXA) scanning in postmenopausal women and men aged > 49 years is recommended. Treatment with calcium and vitamin D supplementation and/or bisphosphonates has been shown to be safe and effective in PLHIVs. However, there is a paucity of data in PLHIV with hormonal therapy. A small, recent study of denosumab, the monoclonal antibody antagonist of RANKL, which is increased in PLHIV, suggested that it was safe and effective in men who are HIV positive [19]. If available, the use of tenofovir alafenamide (TAF) instead of TDF-containing regimens, either as initial treatment to prevent bone loss or as switch therapy in the setting of low bone density, is recommended due to its relative sparing of bone density compared with TDF [20,21]. 

## 7. Conclusions

Sir William Osler (12 July 1849–29 December 1919) was quoted as professing “He who knows syphilis knows medicine.” The same may be said of HIV. Multisystemic metabolic effects have been associated with the infection itself, from immune response to infection, and with the treatments for these infections; molecularly accelerated aging processes have been associated with the infection. A broad understanding of the underlying pathophysiology and up to date practical knowledge of clinical medicine is required to adequately prevent, diagnose, and manage these metabolic complications of HIV. Despite the development of a toolkit of effective HIV-prevention strategies, in the absence of a vaccine, HIV infections will continue in the foreseeable future. Continued advancement of antiretroviral penetrance and reductions in healthcare inequity will continue to prolong the lives of PLHIVs, and the incidence of these metabolic complications will necessarily increase. It is also likely that similar metabolic complications will be recognized in other increasingly common clinical syndromes, including chronic non-HIV infections and chronic non-AIDS immunocompromise states. 

## Data Availability

Not applicable.
